# Auditory target processing in methadone substituted opiate addicts: The effect of nicotine in controls

**DOI:** 10.1186/1471-244X-7-63

**Published:** 2007-11-06

**Authors:** Bernhard W Müller, Michael Specka, Nicolai Steinchen, Dieter Zerbin, Ernst Lodemann, Thomas Finkbeiner, Norbert Scherbaum

**Affiliations:** 1 Clinic for Addictive Behaviour and Addiction Medicine, University of Duisburg-Essen, Virchowstr. 174, 45147 Essen, Germany; 2 Clinic for Psychiatry and Psychotherapy, Virchowstr. 174, University of Duisburg-Essen, 45147 Essen, Germany; 3 Clinic for Psychiatry and Psychotherapy, Volksgartenstr. 40, 44388 Dortmund, Germany

## Abstract

**Background:**

The P300 component of the auditory evoked potential is an indicator of attention dependent target processing. Only a few studies have assessed cognitive function in substituted opiate addicts by means of evoked potential recordings. In addition, P300 data suggest that chronic nicotine use reduces P300 amplitudes. While nicotine and opiate effects combine in addicted subjects, here we investigated the P300 component of the auditory event related potential in methadone substituted opiate addicts with and without concomitant non-opioid drug use in comparison to a group of control subjects with and without nicotine consumption.

**Methods:**

We assessed 47 opiate addicted out-patients under current methadone substitution and 65 control subjects matched for age and gender in an 2-stimulus auditory oddball paradigm. Patients were grouped for those with and without additional non-opioid drug use and controls were grouped for current nicotine use. P300 amplitude and latency data were analyzed at electrodes Fz, Cz and Pz.

**Results:**

Patients and controls did not differ with regard to P300 amplitudes and latencies when whole groups were compared. Subgroup analyses revealed significantly reduced P300 amplitudes in controls with nicotine use when compared to those without. P300 amplitudes of methadone substituted opiate addicts were in between the two control groups and did not differ with regard to additional non-opioid use. Controls with nicotine had lower P300 amplitudes when compared to patients with concomitant non-opioid drugs. No P300 latency effects were found.

**Conclusion:**

Attention dependent target processing as indexed by the P300 component amplitudes and latencies is not reduced in methadone substituted opiate addicts when compared to controls. The effect of nicotine on P300 amplitudes in healthy subjects exceeds the effects of long term opioid addiction under methadone substitution.

## Background

The P300 component of the auditory evoked potential (ERP) can be derived from the EEG in oddball type experiments when rare task-relevant stimuli are interspersed into frequently presented non-task related stimuli [1]. The P300 component of the auditory evoked potential emerges between 260 ms to 500 ms following the onset of the target stimulus. The P300 is one of the most robust components within ERP research and the amplitude of the P300 component rises with lower objective probability and lower subjective certainty about the occurrence of the target stimulus [2]. The P300 reflects attention dependent target processing. A recent model on the neuropsychological basis of the P300 by Polich [3] suggests that a target stimulus initiates frontal lobe activity in the allocation of attention resources needed to perform the task associated with the target stimulus and the exchange of working memory content. Further processes involve the temporal lobe and the parietal cortex with stimulus representation maintenance mechanisms and subsequent memory storage and updating processes. Therefore, a simple task to press a button upon the rare occurrence of a deviant auditory stimulus evokes the initiation of a set of cognitive functions and their related areas in the brain [4].

Methadone substitution is a treatment for subjects addicted to opiates, mainly to heroin. Methadone is a synthetic opiate agonist at cerebral μ- and κ-opiate receptors. If given in a sufficient dose, methadone prevents opiate addicts from withdrawal symptoms after cessation of heroin use [5]. Methadone is taken orally and has an half-life of about 24 hours, so that usually one single dose per day is sufficient. When given in therapeutic doses to people adapted to opiates, methadone exhibits no noticeable psychotropic effects like sedation or euphoria. Nevertheless, the question emerged whether methadone has deteriorating effects on cognitive functioning. Previous studies found impairments in methadone substituted patients relative to healthy controls with respect to attention, psychomotor speed, working memory and information processing [6-9]. It is difficult, though, to differentiate the effects of methadone from other factors, like long term effects of opiate abuse, effects of former or concomitant use of other drugs like alcohol, cannabis or cocaine, or impairments following breath suppression after heroin overdoses [6,10]. Current studies on cognitive abilities indicate that methadone-treated opiate addicts perform better than untreated opiate users, but worse than abstinent former opiate addicts [8,11,12].

The auditory P300 has been assessed in a sample of detoxified opiate addicts with and without methadone substitution [13]. Compared to controls, both patient groups showed decreased amplitudes and increased latencies of the P300 component. While in this study the P300 component in methadone substituted the group was less impaired, this result points to the hypothesis of methadone substitution acts as a stabilizing factor in attention related target processing. In a study by Kouri et al. [14] opiate addicts were detoxified and then treated with and without buprenorphine, a partial κ-antagonist and μ-agonist. While the P300 component decreased after detoxification in placebo treated subjects, buprenorphine reversed this decrement to the level of non-dependent control subjects. While both of these studies used small sample sizes of seven and ten subjects per group, they indicate that opiate addicts may show reduced P300 amplitudes with or without substitution [13], and that substitution treatment may improve auditory target processing in detoxified opiate addicts [14]. The clinical relevance of these neurophysiological studies is to gain insight into whether opiate addicted patients under substitution treatment show deficits which may impact their ability to return to or to attain more stable socio-cultural and occupational settings.

While nicotine use is highly prevalent in methadone substituted patients, with rates of comorbidity reaching 90%and more [15,16], the effects of nicotine on the P300 component may be confounded with those of opiate addiction. Chronic nicotine consumption has been found to be associated with P300 amplitude reduction using a visual oddball paradigm in a large sample of current smokers when compared to subjects who never smoked or those who had terminated smoking [17]. More recently, Neuhaus et al. (2006), using an auditory oddball paradigm, reported persistent P300 amplitude reduction in smokers and furthermore, even in former smokers [18]. Results from these studies indicate that chronic nicotine consumption is associated with P300 amplitude reduction, which may confound results when comparing P300 results of nicotine dependent opiate addicts to those of non-smoking control subjects.

Here we aimed to assess whether methadone substituted opiate addicts differ from control subjects with regard to the auditory P300 component in a two-tone oddball design. Previous studies on the P300 in opiate addicts used small sample sizes and it remains unclear whether the P300 is unaffected in a larger group of methadone substituted opiate addicts when compared to controls. A second aim was to control for the potentially confounding effect of nicotine consumption in control subjects. Therefore, we compared the auditory P300 component in a group of methadone substituted opiate addicts to control subjects with and without current nicotine consumption.

## Methods

We assessed 47 opiate addicted outpatients under current methadone substitution and 65 control subjects matched for age and gender. The study was conducted in compliance with the ethical principles for medical research involving human subjects according to the Declaration of Helsinki and was approved by the local ethics committee of the medical department of the University of Duisburg-Essen. Patients and control subjects gave written informed consent before study inclusion.

### Subjects

Patients had a mean duration of opiate dependency of 9.5 (5.8) years and were under methadone substitution for 19.2 (17.2) month. Patients mean D-L methadone dose was 92.0 mg (53.3) with a D-L methadone/weight ratio of 1.3 (0.8) mg/kg. Among the 47 Patients, 30 had a concomitant non-opioid drug use at the time of assessment [19]. According to urine analysis at the day of assessment, nine of these concomitant drug users had consumed benzodiazepines or benzodiazepines and cannabis combined, 13 patients had used cannabis only, 8 patients had used cocaine with or without additional cannabis or benzodiazepines. Among the 17 patients without non-opioid drug use, two patients had additional heroin use. Given the comparable opioid receptor binding of methadone and heroin, these two patients were included in the opioid group without additional non-opioid consumption. As evidenced by urine analysis, 65 control subjects had negative drug screenings, four additional controls with positive drug screenings were excluded from analysis. Among the 65 drug-free controls, 30 control subjects were nicotine consumers, 35 were not. All methadone patients were nicotine consumers.

Patients and controls did not differ with regard to age comparing whole groups (t = 0.053, p = 0.96) and subgroups of patients with and without concomitant non-opiate use and controls with and without nicotine consumption (F_[3;108] _= 0.08, p = 0.98). Gender did not differ between whole groups (Chi^2 ^= 0.127, p = 0.72) and subgroups (Chi^2 ^= 1.05, p = 0.78). The amount of patients and controls with and without high-school attendance did not differ statistically (Chi^2 ^= 1.14, p = 0.23). In the analysis of subgroups however, there was a higher amount of non-smoking controls with high-school education (Chi^2 ^= 0.81, p = 0.04).

Patients without concomitant non-opioid drug consumption received a higher dose of methadone than those with non-opioid drug consumption (mg absolute and mg dose/weight-ratio, t = 3.67 and t = 3.46, respectively, p < 0.01). The two patient groups did not differ significantly with regard to years of opiate dependency and duration of methadone substitution. Details are given in Table 1.

**Table 1:** Subject characteristics

	**Methadone substituted opiate addicts**			**Control subjects**		
	**whole group**	**without concomitant non-opioid drug use**	**with concomitant non-opioid drug use**	**whole group**	**without nicotine use**	**with nicotine use**
**N**	47	17	30	65	35	30
**Age**	29.3 (5.6)	28.9 (6.6)	29.6 (5.0)	29.4 (6.1)	29.3 (6.3)	29.6 (5.8)
**Gender (m/f)**	34/13	11/6	23/7	45/20	25/10	20/10
**Education**						
**+ high school**	6	3	3	12	10	2
**- high school**	41	14	27	53	25	28
						
***Clinical characteristics:***						
**Duration opiate dep. (y)**	9.5 (5.8)	9.3 (7.6)	9.5 (4.8)			
**Methadone substitution (month)**	19.2 (17.2)	20.6 (19.7)	18.4 (15.9)			
**D-L methadone (mg)**	92.0 (53.3)	125 (58)	73 (40)			
**D-L methadone weight ratio (mg/kg)**	1.3 (0.8)	1.8 (0.7)	1.1 (0.7)			

### Recruitment and assessment procedure

Subjects took part in a study on cognitive-motor performance and auditory target processing which altogether lasted for 2 days. Methadone substituted patients were recruited from 2 outpatient methadone clinics of a psychiatric hospital and from general practitioners. Control subjects were recruited by advertisements in local newspapers and notices at local job-centers and were financially refunded for participation. Subjects interested in participation were invited if they matched the participating patients with respect to age, gender and educational attainment.

EEG assessments were performed in the morning of the second day of the investigation. Subjects were tested for the absence of alcohol intoxication by means of a breath analyzer and gave an urine sample. Drug use was assessed by urine screening for amphetamines, barbiturates, benzodiazepines, cocaine, cannabis, cocaine, heroin and methadone using fluorescence immunoassay technique (FPIA, [20]). With the FPIA technique, opiates can be verified within 2–3 days after consumption, cocaine within 2–4 days, benzodiazepines within 2–4 days after a single consumption and up to 2–3 weeks after chronic consumption. Cannabis can be verified 1 day after a single consumption and up to 2–3 weeks following chronic consumption. Smoking status was ascertained by self report. Patients received their methadone dose one hour before EEG measurements, so that the peak of methadone blood concentration was reached before the start of the assessment.

### P300 evoked potential recording

Subjects were seated in a reclining chair in an electrically shielded room and were presented randomized auditory standard (86%, 800 Hz, 65 dBh, 50 ms, 10 ms rise/fall time) and target stimuli (14%, 1400 Hz, 65 dBh, 50 ms, 10 ms rise/fall time) with an ISI of 3000 (+/- 500) ms. Subjects had to press a button upon the occurrence of the target stimulus. EEG recordings were conducted between 8 am and 12 am in patients as well as in control subjects. EEG was recorded from 19 electrodes (international 10/20 system, Electrocap Inc.) referenced to linked earlobes and from two additional bipolar EOG channels (horizontal and vertical) using a Siemens EEG-21 amplifier with 0.2–70 Hz band pass filter (24 dB/octave). EEG-Data were digitized at 256 Hz from -100 ms to 900 ms relative to stimulus onsets. Trials exceeding +/- 70 μV in horizontal or vertical EOG were discarded from analysis.

Digitized EEG trials in the time window between -100 ms and 900 ms relative to stimulus onsets were baseline corrected and averaged separately for standard and deviant stimuli using in-house software [21,22]. P300 amplitudes and latencies were derived from 3 midline electrodes Fz, Cz, Pz in the range of 260 ms to 500 ms. The mean number of averaged standard tone sweeps was 288.5 (35.4) in patients and 289.4 (47.5) in controls (p = 0.91). The mean number of averaged target tone sweeps was 41.0 (5.5) in patients and 43.7 (6.2) in controls (p = 0.02). The amount of standard and target sweeps did not differ between groups and subgroups.

### Statistical analysis

Group comparisons regarding continuously measured matching criteria and methadone patients' clinical data were carried out using the independent samples t-test in case of 2 groups and one-way analysis of variance in case of 4 groups. Categorial data were analyzed using the Chi-square test. P300 amplitudes and latencies were analyzed using repeated measures analysis of variance with electrode as within-subject factor and group membership as between-subjects factor. For within-subject tests (group × electrode interactions), epsilon-corrected averaged F-tests (Greenhouse-Geisser) were used. While the 4 subgroups were not parallel with respect to school education, a 4-level measurement of educational attainment (no attainment, secondary school attainment [the German Hauptschul-Abschluss], qualified secondary school attainment [Realschulabschluss], higher qualifications) was included as covariate when subgroup membership was the between subject factor. Results with p < 0.05 were regarded as significant. Statistical analyses were carried out using SPSS v13.0 software.

## Results

### Behavioral data

Patients made 0.45 (SD 1.06) errors of commission and 0.62 (SD 1.68) errors of omission. Controls made 0.22 (SD 0.60) errors of commission and 0.22 (SD 0.62) errors of omission. Patients and controls did not differ with regard to both error types (t-tests with corrections for unequal variances, commission errors t = 1.35 and omission errors t = 1.57 respectively, both p > 0.1). Means of reaction times to target stimuli and the intra-subject standard deviations of reaction times as a measure of their attention dependent reaction time stability are given in Table 2. Patients had non-significantly shorter reaction times than controls (t = 0.69, p = 0.49). Patients and controls did not differ in their reaction time dispersion (t = 0.57, p = 0.57). The comparison of subgroups of patients with and without additional non-opioid drug use and of controls with and without nicotine use revealed significant differences between subgroups with regard to reaction time (F_[3;108] _= 2.85, p = 0.041) and reaction time standard deviations (F_[3;108] _= 4.09, p = 0.009). When education was taken into account, significant subgroup differences remained stable only with regard to the reaction time dispersion result (F_[3;107] _= 3.79, p = 0.013). Effects were due mainly to differences between controls: non smoking controls showed lower intra-subject reaction time variability (F_[1;62]_= 5.40, p = 0.023 indicating a higher stability of attention processing over time.

**Table 2:** ERP P300 amplitude, latency and reaction time data

	**Methadone substituted opiate addicts**			**Control subjects**		
	**whole group**	**without concomitant non-opioid drug use**	**with concomitant non-opioid drug use**	**whole group**	**without nicotine consumption**	**with nicotine consumption**
	mean (sd)	mean (sd)	mean (sd)	mean (sd)	mean (sd)	mean (sd)
**P300 Amplitude (μV)**						
Fz	23.3 (14.3)	22.5 (14.1)	23.8 (15.1)	25.1 (13.3)	30.9 (10.9)	18.3 (12.9)
Cz	27.6 (14.6)	26.5 (15.5)	28.3 (14.2)	26.9 (15.5)	34.2 (12.8)	18.4 (14.1)
Pz	33.4 (12.3)	32.9 (14.4)	33.7 (11.1)	31.7 (14.5)	37.7 (12.7)	24.8 (13.5)
						
**P300 Latency (ms)**						
Fz	327 (36)	337 (39)	322 (34)	335 (20)	331 (18)	340 (21)
Cz	333 (43)	339 (42)	330 (45)	335 (23)	332 (24)	339 (22)
Pz	339 (40)	348 (33)	333 (44)	339 (29)	339 (26)	338 (32)
						
**Reaction Time (RT)**						
RT (ms) between subjects	409 (97)	442 (87)	390 (98)	422 (101)	397 (93)	450 (104)
Standard deviation of RT within subjects (ms)	76 (29)	87 (34)	70 (24)	73 (29)	64 (17)	83 (34)

### P300 amplitude data

Means and standard deviations of P300 amplitude data are given in Table 1. A multivariate analysis of variance with electrode position (Fz, Cz, Pz) as within factor and group (patients, controls) as between factor indicated no differences in P300 amplitudes for the group main effect (F_[1;109] _= 60.0, p = 0.72) or the group × electrode interaction (F_[1.6;168.5] _= 1.55, p = 0.22).

**Figure 1:**
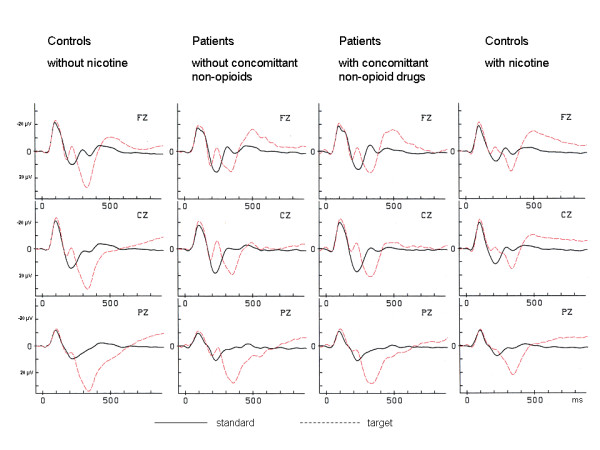
**P300 ERP grand average plots**. ERP grand average plots at electrodes Fz, Cz and Pz (μV) in subgroups of methadone substituted patients with and without additional non-opioid drug use and in controls with and without nicotine use. Note: according to electrophysiological convention negative evoked potential data are shown upwards.

**Figure 2:**
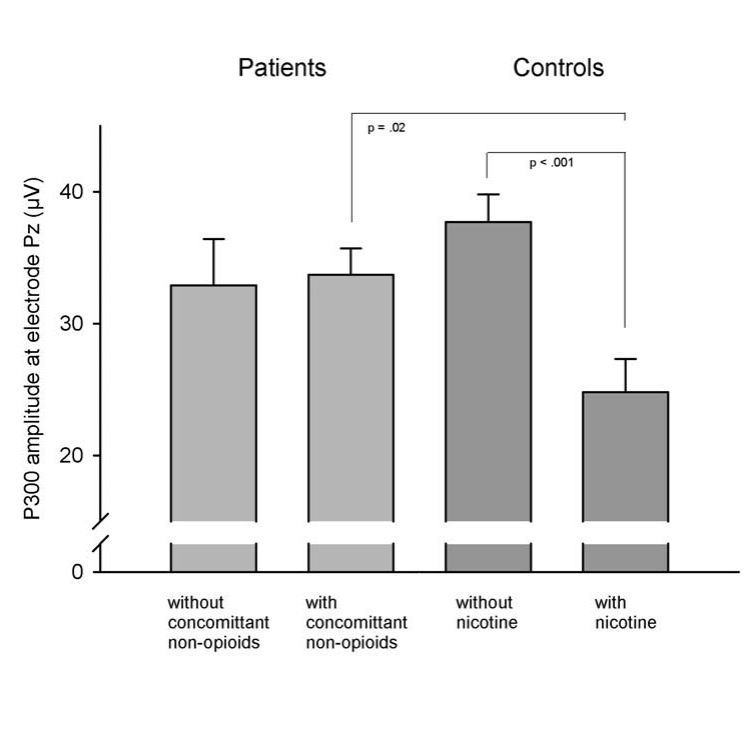
**Mean P300 amplitude at electrode Pz**. Mean P300 amplitudes and standard error bars at electrode Pz (μV) in patients with and without concomitant non-opioid use and in control subjects with and without nicotine use.

The effect of nicotine in controls (with and without) and the effect of additional non-opioid drug use was assessed in a repeated measurements ANOVA with group as between factor and electrodes (Fz, Cz, Pz) as within factor, see Figure 1. This analysis revealed a significant group main effect (F_[3;107] _= 5.07, p = 0.003) but no significant electrode × group interaction (F_[4.6;164.1] _= 0.79, p = 0.57) when controlling for the effect of education (F_[1;107] _= 3.4, p = 0.07). Group effects were further evaluated in group × electrode follow up analyses. Concomitant non-opioid drug use versus no concomitant drug use had no effect on P300 amplitudes (main effect group: p = 0.69, group × electrode interaction p = 0.88). Among controls, nicotine consumption had a major impact on P300 amplitudes with a significant group effect (p < 0.001) but no significant electrode × group interaction. Control subjects without nicotine consumption were not significantly different from patients with (p = 0.20) or without (p = 0.09) concomitant non-opioid drug use. Control subjects with nicotine consumption differed significantly from patients with concomitant non-opioid use (p = 0.02) but not from those without (p = 0.10). We found no significant group × electrode interactions in any of these analyses, see Figure 2.

### P300 latency data

Means and standard deviations of P300 latency data are given in Table 1. A multivariate analysis of variance with electrode position (Fz, Cz, Pz) as within factor and group (patients, controls) as between factor indicated no differences in P300 latencies for the group main effect (F_[1;109] _= 0.09, p = 0.77) or the group × electrode interaction (F_[1.4;135.4] _= 2.29, p = 0.12).

The multivariate analysis of variance with 4 subgroups as between factor and electrodes (Fz, Cz, Pz) as within factor revealed no group main effect (F_[3;107] _= 1.45, p = 0.23) and no significant electrode × group interaction when controlling for education (F_[1;107] _= 0.04, p = 0.85]. Because of the lack of significant effects in the main analyses, we computed no additional follow-up tests.

### P300 data and methadone patients' clinical characteristics

Within the patient group, there were no statistically significant correlations between length of opiate addiction, length of methadone treatment, methadone dose or methadone dose per kg bodyweight on the one hand, and P3 amplitudes or P3 latencies on the other hand (all Pearson-coefficients -0.2 < r < 0.2, all p > 0.1).

## Discussion

The comparison of methadone substituted opiate addicts with a group of matched control subjects revealed no significant group differences with regard to P300 amplitudes and latencies indicating no overall differences between patients and controls. In the analysis of subgroups, nicotine use in control subjects was associated with reduced P300 amplitudes and patients P300 amplitudes were in between those of smoking and non-smoking controls. Additional non-opioid drug use in patients had no significant impact upon P300 amplitudes or latencies. In the comparisons of controls with and without nicotine with patients with or without additional non-opioid drug use, only the comparison of patients with non-opioid drug use with smoking controls revealed significant differences with regard to P300 amplitudes. Here, patients with additional non-opioid drug use had significantly higher P300 amplitudes when compared to smoking controls. Latencies did not differ between groups and subgroups. With regard to the first aim of our study we found no attenuation of P300 amplitudes or latencies between patients under methadone substitution treatment and control subjects. With regard to the second aim of our study we found a significant effect of nicotine in control subjects.

Detoxification in opiate addicts has been associated with lowered P300 amplitudes when compared to controls and substitution treatment has been shown to increase P300 amplitudes [13,14]. Regarding cognitive performance, patients under methadone treatment tend to show lower performance in measures of attention, memory and executive function but not in simple reaction time tasks when compared to controls [8,23]. However, methadone treatment has been shown to improve memory and psychomotor speed [11]. In our study, patient's P300 amplitudes were in between those of the two control groups. While all patients in our study were smokers, it may be suggested that methadone treatment may have heightened P300 amplitudes to some degree over those of nicotine using control subjects.

With regard to control subjects, we found significant P300 amplitude differences in the comparison of healthy subjects with and without nicotine use. Nicotine users showed largely reduced P300 amplitudes at the three electrode sites under investigation. Methadone substituted patients did not differ with regard to concurrent non-opiate drug use and did not differ significantly from control groups. Patients P300 amplitudes were in between controls with and without nicotine use. Latencies of the P300 component did not differ between groups. With regard to behavioral data, healthy subjects without nicotine use showed a low intra-subject reaction-time variability.

Acute nicotine effects have been shown to have positive effects in experimental studies on learning and memory functions in animals as well as in humans [24]. With regard to the visual P300, amplitude increases [25] and latency decreases [26] have been reported with acute nicotine use. In a study on the auditory P300, amplitudes have been shown to be decreased in smokers after several hours of abstinence and to be normalized after smoking [27]. In the Houlihan et al. (1996) study however, neither amplitudes nor latencies were affected with smoking in subjects under short time nicotine abstinence [26].

In contrast to acute effects, chronic nicotine use has been shown to be associated with lower cognitive performance. Cognitive performance worsened with nicotine abstinence especially in those subject to maternal nicotine use during pregnancy [28,29]. The detrimental effects of chronic smoking on the P300 have been established in two large scale studies demonstrating amplitude reductions with regard to the auditory [18] and the visual P300 [17]. The sample size in the Anokhin study was large and the authors controlled their results for a couple of additional confounding factors. Acute nicotine use remained a predictor of lowered P300 amplitudes and only family density of alcohol dependency emerged as another independent predictor of P300 amplitude. The effects of current alcoholism and drug dependence effects vanished after controlling for nicotine use [17]. While the effect of sustained nicotine use on the P300 is not new, our study demonstrates, that nicotine use has to be controlled for in studies on cognitive function in patient samples. A higher proportion of nicotine use in patient groups as compared to controls may act as a confound, indicating differences between patient and control samples which in fact may be due to differences in nicotine use.

While in our study P300 amplitudes of substituted, smoking opiate addicts did not differ from smoking or non-smoking controls, nicotine had a substantial effect on the P300 in controls. However it remains unclear, whether chronic nicotine reduces P300 amplitudes or whether subjects with lower P300 amplitudes are more prone to nicotine and use it as some form of self medication. Together with the hypothesis, that opiate and methadone use may increase P300 amplitudes, our data support the notion that effects of nicotine, opiates and effects of additional predisposing factors interact in some complex form [17]. While the interaction of these factors can not be modeled within our data, further studies, assessing nicotine and opiate withdrawal as an experimental variable in smoking opiate dependent patients, will be needed to investigate the differential contributions of nicotine and opiate use in these patients.

Taken together, the results of our study suggest that the effect of nicotine among healthy controls exceeds the effect of concurrent non-opioid drug use among methadone substituted opiate addicts. Low P300 amplitudes and high reaction time variability point to decreases in auditory target processing in subjects with current nicotine use. The fact that we found no electrode × group interactions indicates that the effects of nicotine may be a more general one and not be related specifically to frontal or parietal sources of the P300.

When comparing our data to those of previous studies, we find that our P300 amplitudes are considerably high [30,31]. As could be expected in an auditory oddball paradigm with an active response to target stimuli, amplitudes increased from electrode Fz to Pz. A number of factors may have contributed to high P300 amplitudes in our study: A low target probability of 14%, a long inter-stimulus interval of about 3000 ms, the assessment of subjects during morning hours and the assessment of a group of young adults may have added in their effect upon amplitudes in our study [30,31].

The clinical relevance of altered P300 amplitudes follows the relevance of the P300 as an indicator of neuronal activity related to cognitive processing [32-34]. Starting with McCarthy et al. (1997) a number of studies assessed the equivalent of the P300 component with functional MRI [35] and showed that even a simple task which requires subjects to press a button or to count the occurrence of a rare target stimulus activates a complex network of neuronal generators, with many of related to attention processing. Functional imaging studies will have to further assess the effects of nicotine and nicotine deprivation on cognitive function which up to now indicate effects on dorsolateral prefrontal cortex [36,37], which has been identified as part of the generators of the P300 in fMRI experiments [4,35].

The clinical conclusion of our study is that, as far as the P300 indexes relevant parts of basal cognitive processes related to attention, context updating and memory processes, patients under methadone substitution treatment are not impaired. Some of the patients, those with additional non-opioid drug use, even show larger P300 amplitudes than smoking controls. This result adds to the evidence that methadone substitution treatment may be a reasonable strategy to give these patients a basis to return to or to attain more stable socio-cultural and occupational settings.

## Conclusion

In summary, the results of our study revealed that P300 latencies and amplitudes in methadone substituted opiate addicts are within the range of control subjects variation. With regard to reaction times we only found an effect of improved intra-subject reaction time variability in controls without nicotine use. Therefore our data do not support the notion of a lowered auditory target processing in methadone substituted opiate addicts, especially when compared to smoking control subjects. A surprising result of our study was that the effect of nicotine in healthy controls exceeded the effect of additional non-opiate use among patients and the patient/control subject group effects. With regard to the P300 as an indicator of attention dependent information processing, our results indirectly indicate that nicotine may have more detrimental effects than a history of opiate addiction when under methadone treatment. Therefore nicotine use has to be taken into account in further studies on cognitive performance and information processing in psychiatric research. Further studies are needed in order to disentangle the differential effects of nicotine and opiates in patients with substance dependency.

## Competing interests

The author(s) declare that they have no competing interests.

## Authors' contributions

TF and EL designed the study. EL, TF, MS and DZ recruited and assessed patients and controls. BWM, MS and NS analysed the data. BWM, MS and NS interpreted the data and prepared the manuscript. All authors have read and approved the final version of the manuscript.

## Pre-publication history

The pre-publication history for this paper can be accessed here:


